# Major food sources of calories, added sugars, and saturated fat and their contribution to essential nutrient intakes in the U.S. diet: data from the national health and nutrition examination survey (2003–2006)

**DOI:** 10.1186/1475-2891-12-116

**Published:** 2013-08-08

**Authors:** Peter J Huth, Victor L Fulgoni, Debra R Keast, Keigan Park, Nancy Auestad

**Affiliations:** 1 PJH Nutritional Science, N5001 565th Street, Menomonie 54751, WI, USA; 2 Nutrition Impact, LLC, 9725 D Drive North, 49014 Battle Creek, MI, USA; 3 Food & Nutrition Database Research, Inc, 1801 Shadywood Lane, 48864 Okemos, MI, USA; 4 Nutrition Research, Dairy Research Institute, 10255 West Higgins Road, Suite 900, 60018 Rosemont, IL, USA; 5 Regulatory Affairs, Dairy Research Institute, 10255 West Higgins Road, Suite 900, 60018 Rosemont, IL, USA

**Keywords:** Foods, Nutrients, Nutrition and health, NHANES, Disaggregation

## Abstract

**Background:**

The risk of chronic disease cannot be predicted simply by the content of a single nutrient in a food or food group in the diet. The contribution of food sources of calories, added sugars and saturated fat (SFA) to intakes of dietary fiber and micronutrients of public health importance is also relevant to understanding the overall dietary impact of these foods.

**Objective:**

Identify the top food sources of calories, added sugars and SFA in the U.S. diet and quantify their contribution to fiber and micronutrient intakes.

**Methods:**

Single 24-hour dietary recalls (Day 1) collected from participants ≥2 years (n = 16,822) of the What We Eat in America, National Health and Nutrition Examination Survey (WWEIA/NHANES 2003–2006) were analyzed. All analyses included sample weights to account for the survey design. Calorie and nutrient intakes from foods included contributions from disaggregated food mixtures and tabulated by rank order.

**Results:**

No one food category contributes more than 7.2% of calories to the overall U.S. diet, but half of the top 10 contribute 10% or more of total dietary fiber and micronutrients. Three of the top 10 sources of calories and SFA (beef, milk and cheese) contribute 46.3% of the calcium, 49.5% of the vitamin D, 42.3% of the vitamin B_12_ as well as other essential nutrients to the American diet. On the other hand, foods categorized as desserts, snacks, or beverages, contribute 13.6% of total calories, 83% of added sugar intake, and provide little or no nutritional value. Including food components of disaggregated recipes more accurately estimated the contribution of foods like beef, milk or cheese to overall nutrient intake compared to “as consumed” food categorizations.

**Conclusions:**

Some food sources of calories, added sugars and SFA make major contributions to American dietary fiber and micronutrient intakes. Dietary modifications targeting reductions in calories, added sugar, or SFA need to take these key micronutrient sources into account so as not to have the unintended consequence of lowering overall dietary quality.

## Background

The health promoting quality of the overall diet is associated with total daily calorie and nutrient intakes. The Dietary Guidelines for Americans (DGA) provides advice for choosing healthy eating patterns for all Americans, including those who are overweight or obese and at increased risk of chronic diseases [1]. The DGA calls for individuals to maintain a healthy weight by controlling calorie intake, increasing physical activity and to consume nutrient-dense foods and beverages to ensure adequate nutrient intake within calorie needs. In addition, the population is advised to reduce calories from added sugars and limit calories from saturated fats (SFA). However, it is recognized that small amounts of fats and added sugar can be useful for increasing the palatability of nutrient-dense foods. Americans are encouraged also to meet their nutrient needs primarily through foods, which are complex and variable in their content of calories, dietary essential nutrients as well as added sugars or SFA.

While staying within calorie needs and as part of a healthy eating pattern, increasing intakes of potassium, dietary fiber, calcium, and vitamin D from nutrient-dense foods like vegetables, fruits, whole grains, and milk and milk products is recommended [1]. Consumption of these four nutrients is so low across the US population in general that they were identified as “nutrients of concern” by the DGA and worthy of special public health interest. Iron and folate for pregnant women and vitamin B_12_ for older adults are also recognized as nutrients of concern specifically for these populations. Because the vast majority of Americans do not consume recommended intakes of the nutrient-dense foods from the basic food groups [2], it is important that dietary advice to reduce intake of certain foods and food components not have the unintended consequence of leading to reduced intake of dietary essential nutrients, including nutrients of concern.

National dietary surveillance through the What We Eat in America (WWEIA), National Health and Nutrition Education Survey (NHANES) provides a way to examine eating patterns and their impact on calorie and nutrient intakes across the U.S. population. Various approaches to food group classifications [3-8] and diet modeling [8,9] have been published. Food groupings based on foods as they are consumed (e.g., chili; Mexican mixed dishes; pizza; soups) compared to those that include components (e.g., cheese; chicken; vegetables; grains; fats and oils; etc.) of disaggregated mixtures produce different pictures of the population’s food and nutrient intakes. The DGA used the National Cancer Institute’s (NCI) categorization system [10], which creates 96 “specific food” categories using a type of “as consumed” methodology. This approach estimates nutrient contributions from entrées and foods eaten alone, however, it does not assess the total nutrient contributions from foods like cheese that can be consumed both alone and as ingredients in an entrée (e.g., burrito; lasagna; omelet; cheeseburger; pizza; etc.) or other mixed dish. Under the NCI system, cheese is categorized as regular cheese or reduced fat cheese [10]. The cheese group, however, does not include cheese also eaten as a component of 14 other categories of culturally diverse mixed dishes [10]. Other systems [3,4,7,11] of food classification disaggregate foods using recipes for mixtures to provide an estimate of the total nutrient contributions from food sources. The disaggregation (also called “as ingredient”) approach was selected by the European Food Safety Administration to harmonize food classification systems across the European Union [12].

This paper builds on previously published food and nutrient intake data by examining the contributions of the top ten food sources of calories, added sugar, and SFA to the population’s intakes of the nutrients of concern as well as other essential nutrients using the disaggregated approach to classify food sources. The present study expands on the study by Reedy and Krebs-Smith (2010) that examined the top five food sources of energy, solid fats and added sugars, referred to as major food sources, from foods consumed by children and adolescents using the as consumed food classification approach [6]. The hypothesis for the present study was that top food sources of calories, added sugars and SFA in current eating patterns make major contributions to intakes of the nutrients of concern (i.e. potassium, dietary fiber, calcium, and vitamin D) as well as other dietary essential nutrients.

## Methods

The analytical and statistical methods used for grouping foods, disaggregation of recipes and ranking top food sources have been published previously [7,13] and will be described here in brief.

### Data source

Data from WWEIA, the dietary component of the 2003–2004 and 2005–2006 NHANES were used in this study [14,15]. NHANES is a nationally representative, ongoing data collection initiative conducted by the Centers for Disease Control and Prevention, National Center for Health Statistics (NCHS), and the dietary component is conducted by the United States Department of Agriculture (USDA). The purpose of NHANES is to collect data on the health and diet of the non-institutionalized civilian population in the United States. The study design is a stratified, multistage probability sample based on selection of counties, blocks, households, and the number of people within households.

### Samples and dietary intake

Data from adults, adolescents and children ≥2 years of age (n = 16,822) participating in the WWEIA/NHANES conducted in 2003–2004 and 2005–2006 were combined for these analyses. Food intake data were obtained from in-person 24-hour dietary recall interviews administered using an automated multiple-pass method [16]. Survey participants 12 years and older completed the dietary interview on their own; children 6 to 11 years old were assisted by an adult; parents/guardians reported for children younger than 5 years of age. Food and nutrient intake data from the first dietary recall was used for these analyses. Data judged incomplete or unreliable by USDA Food Surveys Research Group staff were excluded from analyses, as were those from pregnant and/or lactating females (n = 711). Detailed descriptions of the dietary interview methods are provided in the NHANES Dietary Interviews Procedure Manual [17].

### Food groupings and composition

The United States Department of Agriculture (USDA) Dietary Sources of Nutrients (DSN) database was used to define food groups [18]. The more than 130 DSN food groups were collapsed into 51 categories that were more consistent with food groups defined by the USDA Food Surveys Research Group [19,20], similar to those listed by Cotton et al. [3] and the 2005 Dietary Guidelines Advisory Committee report [21]. These are the same food groups previously published by our group [7].

Disaggregated ingredients of recipes for mixtures were assigned to DSN food groups using the USDA Nutrient Database for Standard Reference (SR) food codes [18]. Ingredients were linked to the appropriate food composition databases using the SR-Link file of the Food and Nutrient Database for Dietary Studies (FNDDS 2.0 and 3.0 linked to SR18 and SR20, respectively) [19,20], and recipe calculations were performed to determine proportions of the mixture’s nutrient composition contributed from the disaggregated DSN food groups. Added sugars composition were derived from the USDA MyPyramid Equivalents Database (version 2.0) [22] and any foods not listed were hand matched to similar foods.

### Statistical analyses

The population mean and standard error (SE) of calorie and nutrient intake for those ≥2 years of age from the total diet and from each food group were determined using PROC DESCRIPT of SUDAAN (release 9.0, Research Triangle Institute, Research Triangle Park, NC) using appropriate NHANES weighting factors which adjust for oversampling of selected groups, survey non-response of some individuals, and day of the week when the interview was conducted. Percentages of total calorie and nutrient intakes from each food group were calculated from population average consumption of each food group and tabulated by ranked order similar to our previous publication [7].

## Results

Calories –The mean total calorie intake by persons two years and older in the U.S. was 2,176 kcal/day. Table 1 shows the total calorie contributions from the top ten food sources of calorie intake: ‘cake, cookies, quick bread, pastry, and pie‘ (7.2%) followed by ‘yeast breads and rolls’ (7.1%), ‘soft drinks’ (5.4%),‘beef’ (4.7%),‘crackers, popcorn, pretzels, chips’ (4.7%), ‘cheese’ (4.6%), ‘milk’ (4.5%), ‘candy, sugars, sugary foods’ (4.5%), ‘poultry’ (4.3%), and ‘alcoholic beverages’ (3.7%). While no individual food category contributes more than 7.2% of total daily calorie intake, together, these foods represent slightly over half (50.8%) of the total daily caloric intake from the U.S. diet. With the exception of ‘soft drinks, soda,’, ‘candy, sugars, and sugary foods,’ and ‘alcoholic beverages’, these calorie sources contribute noteworthy amounts of nutrients of concern to the diet (% total daily intake): calcium (53.3%), vitamin D (49.5%), fiber (22.2%), potassium (11.6%), and nutrients that the DGA recommends certain sub-populations increase: vitamin B_12_ (42.3%), iron (25.1%), folate (22.1%). Four of these top calorie sources (‘poultry’, ‘beef’, ‘cheese’, ‘milk’) together contribute almost one-half of the total daily protein (44.5%) while providing 18.2% of total daily calories.

**Table 1:** Contribution of the top 10 calories sources in the US diet to nutrient intakes

**Calories sources**	**% total kcal intake**	**Macronutrients**	**% total intake**	Micronutrients and fiber*	**% total intake**
Cakes, cookies, quick bread, pastry, pie	7.2	Carbohydrates	8.7	Iron	6.2
		Fat	7.7	Folate	5.9
		Protein	2.5	Fiber	5.1
				Vitamin E	6.6
				Thiamin	6.0
Yeast breads and rolls	7.1	Carbohydrates	10.5	Fiber	10.8
		Fat	2.7	Calcium	7.0
		Protein	6.4	Folate	16.2
				Iron	12.4
				Thiamin	14.4
				Niacin	9.9
				Sodium	8.7
				Riboflavin	7.8
				Magnesium	5.9
Soft drinks, soda (includes diet)	5.4	Carbohydrates	11.2		
		Fat	<0.1		
		Protein	0.4		
Beef	4.7	Carbohydrates	<0.1	Vitamin B_12_	18.6
		Fat	7.5	Iron	6.5
		Protein	13.4	Zinc	20.1
				Niacin	8.8
				Vitamin B_6_	8.2
				Phosphorus	6.3
Crackers, popcorn, pretzels, chips	4.7	Carbohydrates	4.7	Fiber	6.3
		Fat	6.3	Vitamin E	9.4
		Protein	2.0	Magnesium	5.3
Cheese	4.6	Carbohydrates	0.5	Calcium	21.0
		Fat	8.9	Vitamin B_12_	6.6
		Protein	8.8	Phosphorus	11.4
				Vitamin A	9.2
				Sodium	7.6
				Zinc	7.5
				Riboflavin	5.2
Milk	4.6	Carbohydrates	3.6	Vitamin D	49.5
		Fat	4.6	Calcium	25.3
		Protein	8.3	Potassium	11.6
				Vitamin B_12_	17.1
				Riboflavin	16.5
				Vitamin A	16.1
				Phosphorus	14.4
				Magnesium	7.9
				Zinc	7.0
Candy, sugars and sugary foods	4.5	Carbohydrates	7.7		
		Fat	2.1		
		Protein	0.7		
Poultry	4.3	Carbohydrates	0.5	Niacin	15.1
		Fat	5.5	Vitamin B_6_	9.1
		Protein	14.0	Phosphorus	6.6
				Zinc	6.0
Alcoholic beverages	3.7	Carbohydrates	1.6		
		Fat	<0.1		
		Protein	0.6		

*Micronutrients and fiber are shown when the food contributes ≥ 5% of the total daily intake. Micronutrients recognized by the 2010 DGA as nutrients of public health concern are calcium, vitamin D, potassium and fiber, and nutrients identified as those to encourage for specific subpopulations are vitamin B_12_, iron and folate. Folate is reported as Dietary Folate Equivalents (DFE).

Added sugars – The mean intake of added sugars by persons two years and older in the U.S. was 83.9 g/day. Table 2 shows that the top 10 food sources of added sugars in the U.S. diet were: ‘soft drink, soda’ (33.0%), ‘candy, sugars, sugary foods’ (19.5%), ‘cake, cookies, quick bread, pastry, and pie’ (14.4%), ‘fruit drinks and ades’ (11.0%), ‘milk desserts’ (5.4%), ‘ready-to-eat cereal’ (3.9%), ‘yeast breads and rolls’ (2.1%), ‘milk drinks’ (1.8%), ‘yogurt’ (1.0%), ‘condiments and sauces’ (0.9%). These 10 food sources account for 93% of added sugars and 68% of the total sugars in the U.S. diet. The top source of added sugar, ‘soft drinks, soda’, contributes one-third of the total daily intake by Americans and the top three sources together account for 66.9% and 45.5% of added and total sugars, respectively. With the exception of ‘fruit drinks and -ades’, which contributed 16.3% of vitamin C, and ‘cakes, cookies, quick bread, pastry, pie’ which provided 5.1% fiber, 6.2% iron and 5.9% folate, the top five sources of added sugars provided no or limited amounts of essential nutrients to the diet. In contrast, ‘ready-to-eat cereal’ and ‘yeast breads and rolls’ accounted for 3.9% and 2.1% of added sugars, respectively. Together these sources contributed to the intake of several nutrients including nutrients of concern: fiber (16.4%), calcium (7.0%), vitamin D (6.2%) and nutrients to increase by certain sub-populations: folate (37.8%), iron (29.4%), vitamin B_12_ (13.9%).

**Table 2:** Contribution of the top 10 sources of added sugars in the US diet to nutrient intakes

**Sources of added sugars**	**Added**	**Total**	Micronutrients and fiber*	**% total intake**
	**sugars**	**sugars**		
	**% total intake**	**% total intake**		
Soft drinks, soda (includes diet)	33.0	21.8		
Candy, sugars and sugary foods	19.5	13.4		
Cakes, cookies, quick bread, pastry, pie	14.4	10.3	Fiber	5.1
			Iron	6.2
			Folate	5.9
			Vitamin E	6.6
			Thiamin	6.0
Fruit drinks and -ades	11.0	8.2	Vitamin C	16.3
Milk desserts	5.4	4.4		
Ready-to-eat cereal	3.9	2.8	Vitamin D	6.2
			Fiber	5.6
			Folate	21.6
			Iron	17.0
			Vitamin B_12_	13.9
			Vitamin B_6_	14.8
			Thiamin	11.6
			Vitamin A	9.8
			Niacin	9.8
			Zinc	9.3
			Riboflavin	9.3
Yeast breads and rolls	2.1	2.7	Fiber	10.8
			Calcium	7.0
			Folate	16.2
			Iron	12.4
			Thiamin	14.4
			Niacin	9.9
			Sodium	8.7
			Riboflavin	7.8
			Magnesium	5.9
Milk drinks	1.8	1.9		
Yogurt	1.0	1.1		
Condiments and sauces	0.9	1.1		

*Micronutrients and fiber are shown when the food contributes ≥ 5% of the total daily intake. Micronutrients recognized by the 2010 DGA as nutrients of public health concern are calcium, vitamin D, potassium and fiber, and nutrients identified as those to encourage for specific subpopulations are vitamin B_12_, iron and folate. Folate is reported as Dietary Folate Equivalents (DFE).

Saturated fatty acids – The mean total SFA intake by persons two years and older in the U.S. was 27.7 g/day and accounted for 11.4% of total calorie intake. Table 3 shows the top 10 food sources of SFA in the U.S. diet: ‘cheese’ (16.5%), ‘beef’ (8.5%), ‘milk’ (8.3%),‘other fats and oils’ (8.2%), ‘frankfurters, sausages, luncheon meats’(6.9%), ‘cake, cookies, quick bread, pastry, pie’ (6.1%), ‘margarine & butter’ (5.8%), ‘milk desserts’ (5.1%), ‘poultry’ (4.2%), and ‘crackers, popcorn, pretzels, chips’ (4.0%). The top 10 food sources represent three-fourths (73.6%) of SFA intake and also two-thirds of the monounsaturated fat (65.1%) and one-half of the polyunsaturated fat (52.1%) intake in the U.S. diet. The top three food sources of SFA: ‘cheese’, ‘beef’, and ‘milk’ contributed one-third of the SFA (33.3%) and 18.8% of the MUFA. Among these top three sources of SFA, ‘milk’ contributed 49% of the vitamin D and 11.6% of the potassium and ‘milk’ and ‘cheese’ together contributed 46.3% of the calcium – all nutrients of concern. ‘Milk’, ‘beef’ and ‘cheese’ together also contributed about 42.3% of the vitamin B_12_ with ‘beef’ contributing 6.5% of the iron intake.

**Table 3:** Contribution of the top 10 sources of saturated fat in the US diet to nutrient intakes

**Top 10 sources of saturated fat**	**SFA**^**a**^	**MUFA**^**b**^	**PUFA**^**c**^	Micronutrients and fiber*	**% total intake**
	**% total intake**				
Cheese	16.5	6.7	1.5	Calcium	21.0
				Vitamin B_12_	6.6
				Phosphorus	11.4
				Vitamin A	9.2
				Sodium	7.6
				Zinc	7.5
				Riboflavin	5.2
Beef	8.5	8.8	1.2	Vitamin B_12_	18.6
				Iron	6.5
				Zinc	20.1
				Niacin	8.8
				Vitamin B_6_	8.2
				Phosphorus	6.3
Milk	8.3	3.3	1.1	Vitamin D	49.5
				Calcium	25.3
				Potassium	11.6
				Vitamin B_12_	17.1
				Riboflavin	16.5
				Vitamin A	16.1
				Phosphorus	14.4
				Magnesium	7.9
				Zinc	7.0
Other fats and oils	8.2	10.1	11.4	Vitamin E	5.5
Frankfurters, sausages, luncheon meats	6.9	7.8	3.3	Sodium	6.7
Cakes, cookies, quick bread, pastry, pie	6.1	9.0	8.8	Fiber	5.1
				Iron	6.2
				Folate	5.9
				Vitamin E	6.6
				Thiamin	6.0
Margarine & butter	5.8	6.0	6.9	Vitamin A	8.2
				Vitamin E	6.5
Milk desserts	5.1	2.1	0.7		
Poultry	4.2	5.8	6.3	Niacin	15.1
				Vitamin B_6_	9.1
				Phosphorus	6.6
				Zinc	6.0
Crackers, popcorn, pretzels, chips	4.0	5.5	10.9	Fiber	6.3
				Vitamin E	9.4
				Magnesium	5.3

*Micronutrients and fiber are shown when the food contributes ≥ 5% of the total daily intake. Micronutrients recognized by the 2010 DGA as nutrients of public health concern are calcium, vitamin D, potassium and fiber, and nutrients identified as those to encourage for specific subpopulations are vitamin B_12_, iron and folate. Folate is reported as Dietary Folate Equivalents (DFE).

^a^Saturated fatty acids (SFA) include 4:0, 6:0, 8:0, 10:0, 12:0, 14:0, 16:0, and 18:0.

^b^Monounsaturated fatty acids (MUFA) include 16:1, 18:1, 20:1, and 22:1.

^c^Polyunsaturated fatty acids (PUFA) include 18:2, 18:3, 18:4, 20:4, 20:5n-3 (EPA), 22:5n-3 (DPA), and 22:6n-3 (DHA).

Disaggregated food mixtures – Among the top 10 sources of calories, added sugars and SFA, some foods had a higher ranking once recipes were disaggregated compared to how they were ranked in the DGA using the “as consumed” method. These foods included beef, poultry, cheese and milk. This difference in ranking reflects how often the food is consumed alone versus how often it is consumed in mixed dishes. A comparison of calorie contributions when analyzed as “disaggregated” versus “as consumed” found that of the total daily amount consumed, about 55% of beef, about 85% of poultry and about 46% of cheese were consumed alone and about 79% of milk was consumed as a beverage or with cereal. The remainder of the time (to a total of 100%), these foods were consumed as a food component in mixed dishes.

## Discussion

This analysis of NHANES 2003–2006 data using the food disaggregation approach shows that some of the major sources of calories, added sugars, and SFA in the US diet are also major sources of dietary essential nutrients including nutrients that are underconsumed. That said, three of the top 10 sources of calories, including ‘soft drinks, soda,’ ‘candy, sugars, and sugary foods,’ and ‘alcoholic beverages’ contribute calories but have virtually no nutritional value, while the other calorie sources, including beef, poultry, milk, cheese, and baked goods are major sources of nutrients of concern and other essential nutrients. The top five sources of added sugars account for 83% of the population’s added sugar intake but with few exceptions, they provide little or no nutritional value. In contrast, the top three sources of SFA (cheese, beef, and milk) contribute more than 40% of the vitamin B_12_, almost half of the vitamin D and calcium, and are major sources of other essential nutrients to the American diet.

The DGA’s “as consumed” listings of top sources of calories, added sugar, and SFA tell us what foods Americans are putting on their plates that are contributing to high intake of these food components [1]. This information is useful to help consumers identify healthier forms of these foods or to avoid foods with little or no nutritional value. But, in the case of foods that can be eaten by themselves or as a part of mixed dishes, information from a disaggregated approach gives insight into an individual food’s relative contribution to intakes of added sugars and/or SFA as well as essential nutrients to the American diet. For example, compared to DGA rankings, the contribution of beef to SFA intake is actually emphasized by the disaggregated approach as is its importance to the population’s zinc (20.1%) and vitamin B_12_ intake (18.6%). This additional insight can help enable informed choices; e.g., choosing leaner beef rather than eliminating beef from the diet with associated reductions in intakes of certain essential nutrients.

Reduction of total calorie intake for weight loss requires a broad and balanced approach because no one food category makes a large impact on total calories. The food categories with the largest contribution to calorie intake as listed in the DGA are grain-based desserts (6.4% of the total caloric intake) and in the present analyses are ‘cakes, cookies, quick bread, pastry, pie’ (7.2%). But, the present analysis reveals also that three categories (‘soft drinks, soda,’ ‘candy, sugars and sugary foods,’ and ‘alcoholic beverages’) contribute 13.6% of total calorie intake (296 kcal/day) and provide little to no other nutritional value. Reducing intake of these foods could greatly reduce population caloric intake without compromising the overall nutritional quality of the diet.

The predominance of foods providing empty calories is readily apparent in the added sugars analysis. Given the disaggregated food approach in the present study, slightly higher estimates of empty calories are provided by the top five sources of added sugar (83.3%) when compared to the foods listed in the DGA, which are based on the foods as consumed approach (71.7%). The most notable nutrient-dense food in this list, ready-to-eat cereals, contributes only 3.9% of the total added sugar intake while providing 6-22% of 11 different vitamins and minerals to the diet of Americans. Recommending healthier ready-to-eat cereals may be an effective means of increasing intakes of nutrients of concern like fiber, but may lead to only modest reductions to the overall intake of added sugars.

In sharp contrast to the added sugars results, while the top three sources of SFA (cheese, beef, and milk) provide a third of dietary SFA, they also contribute 49.5% of vitamin D, 46.3% of calcium, 42.3% of vitamin B_12_ and 11.6% of the potassium as well as a host of other nutrients to the diet of Americans. The DGA recommends consuming less than 10% of calories from SFA, which is about a 15% reduction from the current 11.4% of calories. This recommendation is based primarily on the role of SFA in increasing LDL cholesterol, which is linked to increased risk for cardiovascular disease [23,24]. However, not all food sources of SFA are the same. Different fatty acid chain lengths have different biological effects, and other non-fatty acid nutrients contained within specific foods also play a role in modifying disease risk (3). Replacing SFA with PUFA, for example, significantly reduces cardiovascular disease risk, whereas the evidence for replacing SFA with carbohydrate or MUFA is less consistent and robust, suggesting that lowering risk may be more strongly related to increased intakes of PUFA rather than decreased SFA [25-27]. Evidence that substituting the omega-6 PUFA, linoleic acid, for SFA may not be beneficial points to the potential for differential effects of specific PUFA [28]. Furthermore, reliance on the level of a single lipid nutrient (SFA) in a food and a single plasma biomarker (LDL-C) may not adequately characterize the cardiovascular impact of complex foods that contain, in addition to SFA, multiple nutrients and other bioactive components that reduce CVD risk. For example, intake of milk and milk products is associated with a reduced risk for CVD despite being a major contributor to SFA intake [1]. Thus, other components in milk and milk products, such as calcium, potassium, magnesium, protein (whey, casein), and vitamins D and B_12_ may confer favorable cardiovascular effects [29-33].

The 2010 Dietary Guidelines Advisory Committee (DGAC) report through its evidence based review concluded that not all SFA have the same effect on disease risk, noting that fat from dairy products is an area that requires further study [34]. The report indicated that consumption of milk products may not have predictable effects on blood lipids and future research should examine the role of dairy products in modulating lipid profiles, noting that bioactive components that alter serum lipid levels may be contained in milk fat. The report also states that evidence to date does not suggest that high-fat dairy products are more likely than low-fat dairy products to induce metabolic syndrome.

More frequent consumption of dairy products, vegetables, fruits, and whole grains is recommended to increase intakes of potassium, dietary fiber, calcium, and vitamin D [1]. The DGA recommends preferentially choosing lean meat and poultry and low-fat and fat-free dairy products, including milk, cheese and yogurt, over higher fat forms to help balance calorie intakes. The widespread availability of low-fat and fat-free milks, however, has not offset the overall decline in milk consumption since 1980 (−21%) and the even larger decline in whole milk consumption alone (−65%) [35]. An Australian study of the dietary consequences of recommending lower-fat dairy foods to overweight adults found men decreased their overall intake of dairy foods significantly, rather than switch to lower fat versions [36]. It is not well understood what role the amount of milk fat plays in maintaining or increasing milk consumption among those with a preference for higher fat milk and encouraging milk consumption among those who infrequently consume milk products.

While the DGA recommends mainly choosing lower-fat cheeses, achieving flavor, texture, color, and other attributes comparable to full-fat versions is challenging for cheese manufacturers, particularly at the greater than 50% reductions in fat [37-41] needed to label cheese as low-fat or fat-free. Low-fat cheddar, for example, must contain 80% less fat than its full-fat counterpart to meet federal labeling requirements. Consumers are discerning and acceptance of lower-fat cheeses can be poor, even when differences are small. Consumer acceptance of reduced-fat cheese, which requires a 25% reduction in fat, has seen greater success than low-fat and fat-free forms of cheese [42,43].

## Conclusions

Overall, the present study determined the contributions of the top food sources of calories, added sugars and SFA to intakes of nutrients of concern and other micronutrients in the U.S. diet using a disaggregated food categorization approach to analyze the NHANES 2003–2006 dietary data. While foods like desserts, snacks and some beverages are major contributors to American consumption of calories and added sugar, these foods provide virtually no other nutrients. On the other hand, the foods contributing the most to SFA intake are also major contributors to calcium, vitamin D, and vitamin B_12_ intake. Thus, the totality of nutrients, and not solely a single component such as added sugars or SFA should be balanced when making food choices to build a healthy diet. The potential impact of reducing or eliminating food sources of saturated fat or added sugars that are also major sources of nutrients in the American diet without substituting lower-fat versions or suitable alternates could result in serious unintended nutritional consequences.

## Competing interests

VLF as Senior Vice President of Nutrition Impact, LLC performs consulting and database analyses for various food and beverage companies and related entities. PJH of PHJ Nutritional Science is a nutrition science consultant for various food companies and related entities. DRK of Food & Nutrition Database Research, Inc. performs statistical analyses for Nutrition Impact, LLC, various food and beverage companies and related entities. KP and NA were/are employees of the Dairy Research Institute.

## Authors’ contributions

The authors’ responsibilities were as follows- NA/VLF: designed research, project conception, development of overall research plan, and study oversight; VLF/DK: analyzed data or performed statistical analysis; PJH/NA/KP determined the overall content for the paper; PJH/NA/KP/VLF collaborated on the writing of the manuscript; PJH: had primary responsibility for final content. All authors read and approved the final manuscript.
